# Dynamic causal modeling and Granger causality Comments on: The identification of interacting networks in the brain using fMRI: Model selection, causality and deconvolution

**DOI:** 10.1016/j.neuroimage.2009.09.031

**Published:** 2011-09-15

**Authors:** Karl Friston

**Affiliations:** The Wellcome Trust Centre for Neuroimaging, Institute of Neurology, UCL, 12 Queen Square, London, WC1N 3BG, UK

## Introduction

“These authors and a related commentary (Friston, 2009) concluded that: i) The concepts of temporal precedence and G-causality should not be used in fMRI connectivity analysis (Roebroeck et al. 2011-this issue).”

First, I should say this was not the conclusion I wanted to convey. Second, I apologize for the superficial and dismissive treatment of Granger causality in Friston (2009): I was asked to provide a ‘primer’ that focused specifically on the implications of David et al. (2009) for non-specialists. This focus precluded a balanced discussion of effective connectivity analyses. In contrast, Roebroeck et al. (2011-this issue) provide a comprehensive and compelling treatment of the essential issues; while companion papers (in this section) offer a wider discussion on some of the conceptual and technical issues. In light of these papers, I will limit my comments on Roebroeck et al. to develop or nuance some of the key points they make.

The reservations articulated in Friston (2009) were not about temporal precedence but about the vector autoregressive models (VAR) on which G-causal inference is based. These reservations are technical and formal: Technically, Granger causal analysis (GCA) rests on the theory of Martingales, which requires random fluctuations in the brain to be infinitely ‘rough’ (like white noise). Pedro Valdes Sosa and I (Valdes-Sosa and Friston, 2011) discuss the implications of this elsewhere. The formal reservations are based on the form of VAR or linear stochastic models (LSM), reviewed nicely in Roebroeck et al. These forms can preclude an interpretation of the model parameters in terms of directed connectivity; I deal with this below but start by commenting on two key themes Roebroeck et al. highlight.

## Model comparison and selection

“Exploratory techniques, like GCM, use information in the data to investigate the relative applicability of many models. As such, they have the potential to detect ‘missing’ regions in structural models. Confirmatory approaches, like DCM, test hypotheses about connectivity within a small set of models assumed to be applicable (Roebroeck et al., 2011-this issue).”

This is an important distinction and highlights the fact that DCM is usually called upon, after an exploratory analysis, to answer questions about the connectivity architectures that mediate observed regional responses. These exploratory (whole brain) analyses usually identify co-activated areas (with conventional SPM) or sometimes use pychophysiological interactions (to identify regions whose connections may show task-dependent changes). Granger causality mapping (GCM) can play an important role in this structural model selection. However, DCM is not usually considered as a confirmatory technique, because its primary use is in dynamic model selection. Put simply, DCM is based on model comparison (of the same sort used to infer G-Causality) that allows one to explore model-space. Here, the models differ in their connections and ensuing dynamics. It is now commonplace to explore model-spaces with hundreds of models (e.g., Fairhall and Ishai, 2007, Stephan et al., 2007a). In this sense, DCM is exploratory. In model comparison, models of the same data are compared in terms of their evidence (this is the basis of Bayes-factors or log-odd ratios). The evidence is simply the probability of the data given a model. The twist here is that, in fMRI, the data change with the structural model, which means one cannot compare structural models with different regions (with GCA or DCM). This makes structural model selection necessary and mandates exploratory analyses. However, this only applies to fMRI; in EEG and MEG, all data channels are used and it is perfectly possible (and optimal) to select the structural model (in terms of dipole number and location) using DCM (e.g., Garrido et al., 2008).

“In the model identification stage the parameters in the chosen model class are estimated from the observed data record. In practice, model selection and identification often occur in a somewhat interactive fashion where, for instance, model selection can be informed by the fit of different models to the data achieved in an identification step. (Roebroeck et al., 2011-this issue).”

This is an important point and highlights the distinction between inference on models (i.e., Bayesian model comparison in DCM or inferring G-causality using the accuracy of models with and without a connection) and inference on unknown parameters of any given model. Model comparison is based on the log-evidence for any model, which comprises accuracy (i.e., fit) and complexity (i.e., number of parameters). In one sense, the ‘interaction’ between model selection and identification is part of model comparison; in that a model with low fit will have smaller evidence (Penny et al., 2004, Stephan et al., 2009). Usually, in DCM one optimizes the model by an exhaustive search of model-space (selecting the model with the greatest log-evidence) and then reports the conditional parameter estimates of the best model.

“The bias/variance trade-off in model fitting dictates that over-fitting a finite dataset with too many parameters will lead to poor generalization of model fit to other data sets. Therefore, clear justifiable choices must be made both in the structural model and the dynamical model to keep the number of estimated parameters in a suitable range. (Roebroeck et al., 2011-this issue).”

In fact, proper model comparison (based on the evidence as opposed to just the accuracy or fit) prevents over-fitting for free, because the log-evidence includes a complexity term (Penny et al., 2004). This means the selection of model-space is not constrained by complexity considerations; if a model has too many parameters its complexity will be high and the evidence will fall below a more parsimonious (generalisable) model.

“The incapability of DCM to model signal variations beyond those implied by the exogenous inputs makes its connectivity estimation highly dependent on the exact number and form of the assumed inputs and the form of the structural model (Roebroeck et al., 2011-this issue).”

This is an important point; namely inference on parameters is conditioned on (and depends on) the model. However, this dependency may or may not be greater for deterministic DCMs (as opposed to stochastic DCMs, which model random fluctuations). This is an empirical question that would be addressed using model comparison (i.e., deterministic vs. stochastic formulations of the same DCM).

“Model selection procedures for connectivity should include consideration of more than just a few brain structures…. The clear danger with overly simple structural models is that of spurious influence: an erroneous influence found between two selected regions that in reality is due to interactions with additional regions which have been ignored (Roebroeck et al., 2011-this issue).”

Although an important issue, it is difficult to substantiate general statements of this sort. In fact, conditional estimates of effective connectivity from a full graph (network) are often very consistent with estimates based on subgraphs. This speaks to a common misconception about DCM; namely, that one will get misleading answers if key regions are omitted. This is not the case. Effective connectivity is the ‘effective’ influence one region exerts over another and can be mediated polysynaptically through other (omitted) regions. This means it is perfectly possible (and common practice) to focus on a small number of interconnected regions and use exogenous inputs as surrogates for afferent input from other regions.

## Hemodynamic convolution

“Undoing the effect of hemodynamics on fMRI data (by deconvolution) can be an important tool. However, it is crucially dependent upon assumptions that need to be verified (Roebroeck et al., 2011-this issue).”

This is absolutely right, in a qualified sense: DCM does not deconvolve neuronal activity from hemodynamic signals and then look for coupling among the ensuing estimates; it inverts or fits a full model of distributed neuronal responses that is equipped with hemodynamics (i.e., a hemodynamic convolution). This means conditional correlations among the neuronal (coupling) and hemodynamic parameters are properly accounted for during inference. Note that

“David et al. performed deconvolution operations to obtain estimates of neuronal source signals for their regions of interest to use in G-causality analysis (Roebroeck et al., 2011-this issue).”

They had to do this because the model used by GCA has no hemodynamics. However, this deconvolution is not necessary in DCM; it is implicit in model inversion. Crucially, DCM for fMRI uses regionally specific hemodynamic parameters so that variations in hemodynamic latency can explain away any spurious temporal precedence in observed signals. This is expressed nicely in Roebroeck et al.:

“For instance, if there are delayed coherent variations between variables in the observed data and the hemodynamic model has much more affordance for delays than the neurodynamic model (as is the case in DCM), then the delay will be put into the hemodynamics in the fitting of the model (Roebroeck et al., 2011-this issue).”

This ensures that regional hemodynamic variations do not confound the estimation of neuronal coupling parameters. One might think that one needs to know the exact form of the hemodynamics that link neuronal activity to BOLD signals to estimate neuronal parameters properly. In fact, as evidenced by model comparison of the sort reported in Stephan et al. (2007b), this is not the case: Any hemodynamic model with sufficient degrees of freedom will do; in the sense that the neuronal parameters are largely unaffected by changing the form of the hemodynamic model. This explains the success of conventional convolution models used in whole-brain SPM analyses, which use linear mixtures of basis functions. In brief, the only thing that matters is the generalized convolution kernel of the optimized hemodynamic mapping, not the form or parameterization of its underlying differential equations (of course, this is not the case if one was interested in the physiology of the hemodynamics per se; such as the effects of aging on vascular compliance). In summary, while the detailed physiology of neurovascular coupling is an active area of research and modeling, we already know more than sufficient to model it phenomenologically, in that there is no evidence in fMRI data for more elaborate models (Stephan et al., 2007b); at least to date.

## Vector autoregression and state-space models

“We will argue that, rather, the important distinctions between DCM and GCM are in a deterministic versus a stochastic dynamical model and in the physical interpretation of its variables.…

Nonetheless, it will be informative to compare the class of Linear Stochastic Models (LSM), of which the AR model used in GCM is a special case, with the DCM signal model to see that their crucial differences are actually subtle (Roebroeck et al., 2011-this issue).”

This brings us to the formal problems with linear stochastic or vector autoregression (VAR) models employed by GCA. The critique of GCA in Friston (2009) was not directed at exploratory GCM (Granger causality Mapping) with bivariate models (Goebel et al., 2003, Roebroeck et al., 2005) but reflected our earlier assessment of multivariate VAR models of networks (i.e., with three or more regions; see Harrison et al., 2003 and Fig. 1 in Friston 2009). The problem can be illustrated in terms of the relationship between linear stochastic equations and the equations of motion used by DCM (see Fig. 2 in Roebroeck et al., 2011-this issue). In brief, a (stochastic) DCM can always be formulated as a VAR model, which takes the following form (ignoring exogenous inputs and observables):

$$\begin{array}{c} \dot{z}(t)=Az(t)+\eta (t)\Rightarrow z(t)=Az(t-1)+ɛ(t)\\ A=\exp(A) \end{array}$$

(where *ɛ*(*t*) is a complicated convolution of the innovation *η*(*t*); see Valdes-Sosa et al., 2009). However, the converse is not true. In other words, there is no necessary mapping between the parameters of a VAR model (the autoregression coefficients **A **∈ ℜ^*n *^^× *n*^) and the coupling parameters (effective connectivity *A* ∈ ℜ^*n *^^× *n*^) that mediate the influence of one state over another. In other words, the effective connectivity associated with the VAR coefficients does not necessarily exist; more formally *A* = ln(**A**) ∈ ℜ^*n *^^× *n*^ when, and only when, all the real eigenvalues of **A** are positive. The problems caused by the mapping **A** = exp(*A*) can be illustrated quite simply by considering the coupling among three regions, where there is no connection from the second to the first, e.g.,

$$A=\left[\begin{array}{lll} -1 & 0 & -.5\\ .5 & -1 & -.5\\ .5 & .5 & -1 \end{array}\right]\Rightarrow A=\left[\begin{array}{lll} .3164 & -.044 & -.169\\ .1241 & .3164 & -.212\\ .2121 & .1681 & .2724 \end{array}\right]$$

Critically, the VAR parameters suggest that the first two areas are reciprocally connected; e.g., **A**_12 _= − .044. This means that that tests for the existence of the VAR (G-causal) parameter **A**_12_ do not test for the corresponding directed connection *A*_12_ = 0. This problem becomes greater with longer time intervals (i.e., TR) between observations, which we assume is one, for convenience.

Happily, bivariate models are exempt from this problem, because the VAR coefficient associated with an absent connection is always zero, e.g.,

$$A=\left[\begin{array}{ll} -1 & 0\\ .5 & -1 \end{array}\right]\Rightarrow A=\left[\begin{array}{ll} .3679 & 0\\ .1839 & .3679 \end{array}\right]$$

This admits their qualified use to detect directed pair-wise coupling, as described in Goebel et al. (2003) and Roebroeck et al. (2005).

In short, there is a formal and fundamental divorce between the forms of network models used by DCM and VAR. This divorce becomes even more acute when we consider high order VAR(p) models. These are required when the number of hidden states exceeds the number of observed data points, as in two-state models for fMRI (Marreiros et al., 2008). At this point, we move beyond the issues considered by Roebroeck et al. (2011-this issue) and so I will close, in the hope that the comments above supplement their useful and informed review.
